# Patterns of exon-intron architecture variation of genes in eukaryotic genomes

**DOI:** 10.1186/1471-2164-10-47

**Published:** 2009-01-24

**Authors:** Liucun Zhu, Ying Zhang, Wen Zhang, Sihai Yang, Jian-Qun Chen, Dacheng Tian

**Affiliations:** 1 State Key Laboratory of Pharmaceutical Biotechnology, Department of Biology, Nanjing University, Nanjing 210093, PR China

## Abstract

**Background:**

The origin and importance of exon-intron architecture comprises one of the remaining mysteries of gene evolution. Several studies have investigated the variations of intron length, GC content, ordinal position in a gene and divergence. However, there is little study about the structural variation of exons and introns.

**Results:**

We investigated the length, GC content, ordinal position and divergence in both exons and introns of 13 eukaryotic genomes, representing plant and animal. Our analyses revealed that three basic patterns of exon-intron variation were present in nearly all analyzed genomes (*P *< 0.001 in most cases): an ordinal reduction of length and divergence in both exon and intron, a co-variation between exon and its flanking introns in their length, GC content and divergence, and a decrease of average exon (or intron) length, GC content and divergence as the total exon numbers of a gene increased. In addition, we observed that the shorter introns had either low or high GC content, and the GC content of long introns was intermediate.

**Conclusion:**

Although the factors contributing to these patterns have not been identified, our results provide three important clues: common factor(s) exist and may shape both exons and introns; the ordinal reduction patterns may reflect a time-orderly evolution; and the larger first and last exons may be splicing-required. These clues provide a framework for elucidating mechanisms involved in the organization of eukaryotic genomes and particularly in building exon-intron structures.

## Background

The major function of exons to present mRNA and to code proteins was discovered around 40 years ago [1]. During the last decade there was a breakthrough in understanding the function of introns [2-4]. The intron sequences were once considered to be junk DNA [5], however, people have recently realized that some of them may be functional [6,7]. These DNAs may harbor a variety of elements that regulate transcription, e.g., untranslated RNAs [8] and splicing control elements [9]. Due to their functional properties, at least a fraction of intronic regions are likely to be evolving under the influence of natural selection, mostly purifying selection [7]. In addition, the structural units, the length and the GC content of first exons and introns, are likely to associate with functional elements in large genomes of complex organisms [10]. The varieties of functional elements in introns are revealed to associate with the function of adjacent exons [11]. Therefore, the evolution of the exon-intron structure of eukaryotic genes becomes the emerged topic.

Recent studies have produced data that shed light on the pattern of intron properties, e.g., the variations of length, GC content, ordinal position in a gene (first intron, second intron, and so on) and divergence of intron sequences. Various factors are revealed to influence the intron size [6,12]. For example, the insertion of transposable elements alters the size of introns [13]. Similarly, the frequency and size of deletion events [14] leads to changes in intron size and the presence of regulatory elements and RNA genes influences the length [15]. Alternative splicing can also change intron/exon size [16]. As a result, the factors controlling gene expression and regulation impose a selective constraint on intron size [17]. Correlations among intron divergence, intron ordinal position and intron length were revealed, suggesting that the structure of introns may be under selection as well [7]. However, the relationship between intron length and GC content appears to be complicated. Gazave et al. [7] showed that there was a strong negative correlation among intron length and GC content and divergence in primates, whereas Haddrill et al. [18] found that the class of long introns had higher GC content and lower divergence than that of short introns in fruit fly.

Different from introns, there is little data about the patterns of exon properties or variations of exon-intron architecture. Only a few studies included the basic statistical analyses, such as the distribution of exon length, the average number of exons per gene from eukaryotic model organisms [19], and the chromosomal distributions of exons [20]. Therefore, a systematic investigation of the properties of both exons and introns will provide a framework for understanding the mechanisms determining exon-intron architecture. The availability of multiple, complete eukaryotic genome sequences makes it possible to examine many fundamental evolutionary questions on the genome scale. Here, we performed an extensive analysis of relationships among length, ordinal position, GC content and divergence of both introns and exons on 13 eukaryotic genomes – six mammals, two plant species, two fish species, chicken, fruit fly and worm. We selected these genomic comparisons because they covered a wide range of eukaryotic species.

Our data revealed three consistent patterns, which present in almost all of the genomes we analyzed. Elucidation of these common patterns provides a basis for understanding the factors responsible for organization of the eukaryotic genomes, and for describing the exon-intron architectures.

## Results

### Pattern of intron length and GC content

The complex relationships between intron length and GC content were revealed in human and fly genome [7,18]. A similar analysis for 13 eukaryotic species (Table 1) showed that these patterns were different among species (Additional file 1, Fig. S1). For example, strong negative correlations (decay curve; Additional file 2, Table S1) existed in all six mammalian genomes (human, chimpanzee, dog, cow, mouse and rat; Additional file 1, Fig. S1A), whereas strong positive correlations were present in fly, rice, zebrafish and worm genomes (Additional file 1, Fig. S1C). Clearly, the relationships between intron length and GC content in different species are complicated, which may be associated with isochore structure of genomes. It is well-known that GC-rich isochores of vertebrate have short introns, while GC-poor isochores have very large introns [21]. Indeed, the patterns of intron length against GC content are different in GC-poor and GC-rich regions (Additional file 1, Fig. S1D).

**Table 1:** Statistics of 13 genomes analyzed

Species	Sequences used (Mb)^1^	CDS count^2^	Exon count^3^	Intron count	Average Exon length (bp)	Average Exon GC (%)	Average Intron length (bp)	Average Intron GC (%)
*A. thaliana*	119.2	23488	135697	118838	223.7	44.1	163.7	32.7
*B. taurus*	2434	16829	162223	151199	162.3	52.0	4516.4	46.9
*C. elegans*	100.3	27123	171102	149895	208.4	43.0	334.7	29.1
*C. familiaris*	2445	15960	161238	147118	157.2	50.6	3535.5	46.1
*D. melanogaster*	118.4	8119	43847	40732	370.0	52.7	1530.7	36.5
*D. rerio*	1547	20256	173438	156972	156.2	48.7	2276.4	34.5
*G. gallus*	1032	14367	142329	133690	152.1	48.2	2811.8	42.6
*H. sapiens*	3077	40430	381122	378089	162.7	50.8	5848.7	45.9
*M. musculus*	2644	30546	254605	243458	170.5	51.1	4683.6	46.0
*O. sativa*	372.1	41046	178106	144863	306.0	51.0	396.1	37.7
*P. troglodytes*	3176	30010	296830	287132	156.2	50.7	6003.7	45.3
*R. norvegicus*	2719	22243	195940	186864	172.9	51.1	4406.4	46.3
*T. nigroviridis*	217.3	15455	122253	108901	174.3	54.5	599.5	45.4

Sequences used were all assembled chromosome sequences in the related databases; CDS count represented all known transcripts available in the table "transcript" of Ensembl database (build 44); Exon (or intron) count did not include these in UTR region.

**Figure 1:**
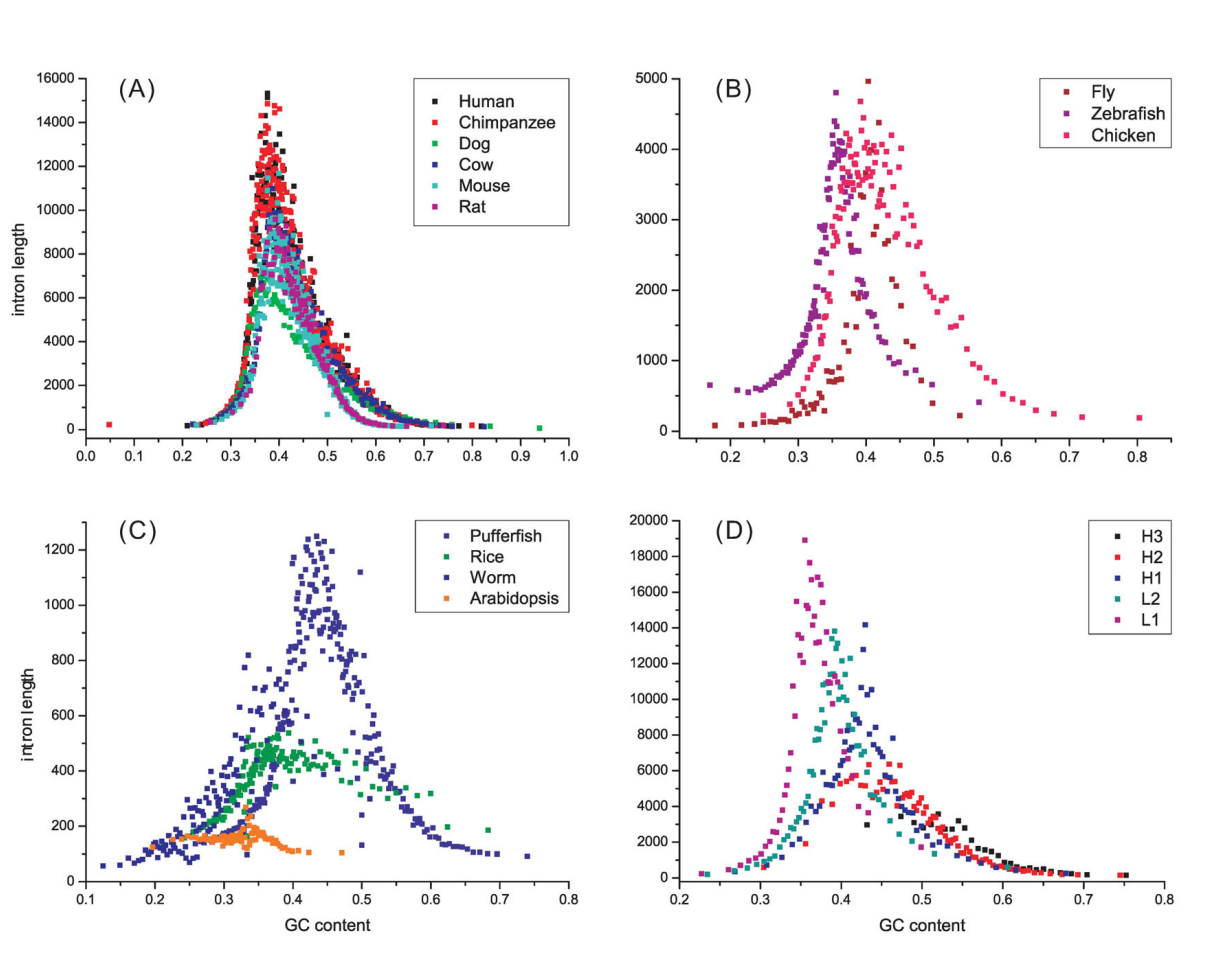
**Variation patterns of introns' length, as a function of GC content**. Each dot contains 1000 introns (A-D). The species in A-C were grouped based on ranges of intron lengths, e.g., up to 16000 bp for mammals. L1, L2, H1, H2 and H3 in D represent five GC-isochore families in human genome [22] which are different in GC content (0~0.37, 0.37~0.41, 0.41~0.46, 0.46~0.53, 0.53~1, respectively). Genes that cover two or more family regions were excluded in D. The x-axis scale in A – D is based on the actual GC content in different genomes.

Interestingly, the complicated relationships between intron length and GC content could be characterized by a curve when the introns were sorted by their GC content from low to high, then the average length of every 1000 introns were plotted against their average GC content (Fig. 1). This simple pattern was significantly present in all species analyzed. The longest introns within a species always have a similar level of GC contents ranging from 35–45% in different species. However, short introns can have either low or high GC contents. Notably, in human genome, this pattern is present not only in the GC-poor isochores but also in GC-rich isochores [22] (Fig. 1D). The consistent pattern observed among various species and isochores suggests that a common mechanism might lead to increased intron length.

**Figure 2:**
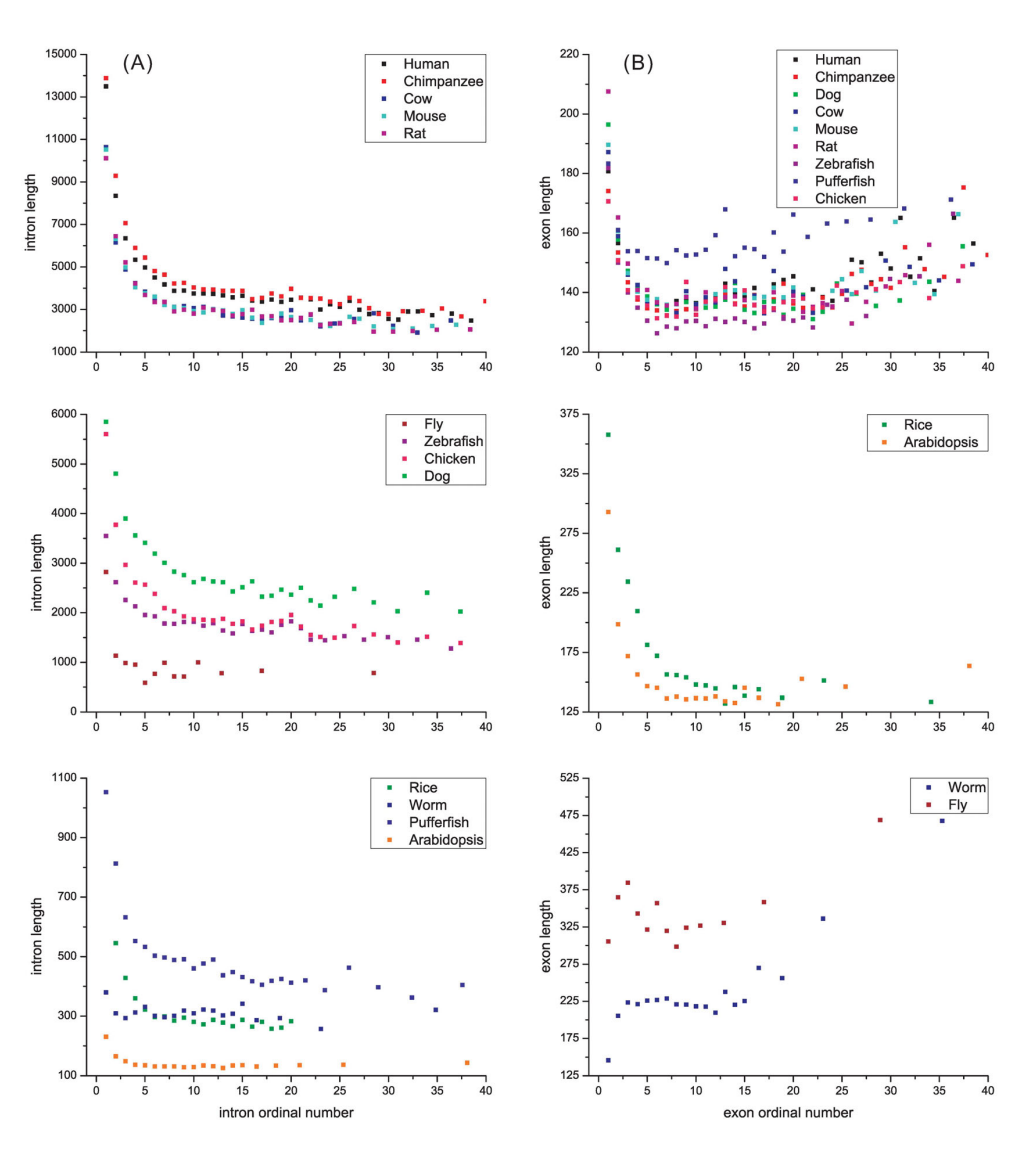
**Variation patterns of intron (A) or exon length (B), as a function of its ordinal number**. Only introns or exons with ordinal numbers ≤ 40 were shown in the figures; each dot contains > 1000 samples.

In addition, intron length is negatively correlated with ordinal position in a gene across all genomes analyzed (*P *< 0.001, Additional file 2, Table S1). Fig. 2A depicts a decay curve formed between intron order and length for all these genomes, however, some of the correlations between intron order and GC content are more complicated (Additional file 1, Fig. S2A). A significant negative correlation (*P *< 0.001) is present in the rice genome as well as in chicken and fly genomes. In mammalian genomes, the GC content of the first intron is significantly higher than the others, and thereafter no perceptible difference was seen. In the other genomes, e.g., worm and zebrafish, no clear variation was found. The results of the systematic investigation on many genomes suggested the intron length was not randomly distributed in the ordinal positions. However, while this supposition may be partly true for the distribution of intron GC content in the ordinal positions, no clear pattern was obtained for the genomes analyzed.

### Pattern of exon length and GC content

The similar pattern observed between intron length and GC content was also observed between exon length and GC content (Additional file 1, Fig. S3). When sorted by exon length from short to long like introns, a negative trend of average GC content of every 1000 introns was present in mammals, although the tendency was not close as that observed for introns (Additional file 1, Fig. S3A). When sorted by exon GC content from low to high, a quadratic correlation significantly fitted the data of all mammalian genomes (*P *< 0.001, Additional file 1, Fig. S3D & Additional file 2, Table S1) as well as all other species (*P *< 0.001, Additional file 1, Fig. S3E-F). In a comparison with Fig. 1 and Additional file 1, Fig. S1, the shapes of curves or lines shown in Additional file 1, Fig. S3 are more variable (not closely correlated), however, our systematic analyses on various genomes suggest a non-random distribution of GC content in exons.

When the last exon, which is the longest one [10], was excluded, the GC content or length was also correlated with the ordinal position, although the correlations were higher in the introns. An observed trend was revealed between exon length and its ordinal position in all species analyzed (Fig. 2B) and a decay curve was observed in mammal, plant and fish genomes (*P *< 0.001 mostly, except for Fly *P *= 0.017). In general, there appears to be a negative tendency between the length and ordinal position of exons. However, a positive correlation was present in worm.

### Co-variation of exon and intron in length and GC content

**Figure 3:**
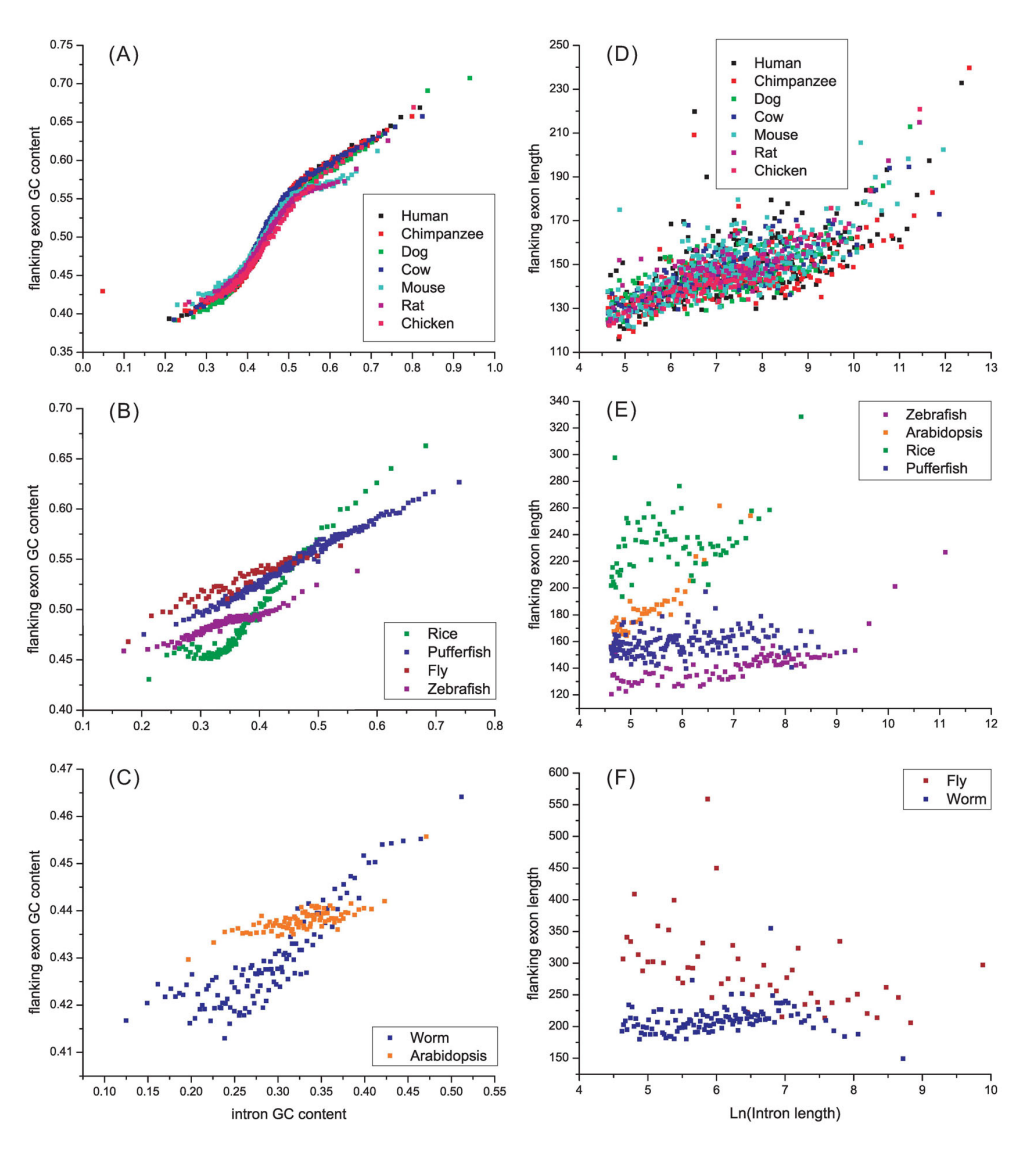
**Variation patterns of exon GC content, as a function of flanking intron GC content (A~D), and exon length, as a function of flanking logarithmical intron length (E~H)**. Each dot contains 1000 introns and 1000 exons (1000 left exons flanking the corresponding introns). Introns used in E~H are > 100 bp.

The similar pattern in the distribution of length and GC content in both introns and exons suggests the existence of co-variation in some of these traits. To test this hypothesis, we calculated all possible correlations among these specific traits between introns and exons, and found that some of traits were correlated. A close positive correlation was found between intron GC content and its flanking exon GC content (or either side, data not shown), and this was present in all analyzed genomes (*P *< 0.001; Fig 3A–C & Additional file 2, Table S1). For example in human genome, the linear correlation is: exon GC-content = 0.6201 × intron GC-content + 0.221; r = 0.977; *P *< 0.001.

The last exon was found to be the longest one in human genes [10]. Our analysis confirmed this phenomenon not only in human genome but also in most of the other genomes analyzed (except for worm and fruit fly). For example, the last exon is about twice longer than the average length of the other exons in human. Therefore, we calculated the correlation between the length of other exons and their flanking introns. When removing the last exon from each of genes analyzed, a positive correlation was present between exon and intron length in all genomes (*P *< 0.001; Fig 3D–E & Additional file 2, Table S1), except for fly and worm (Fig. 3F), where a significantly negative correlation or a random distribution is present (P < 0.001). These results suggest that a longer exon is generally followed by a longer intron. It is noteworthy that our results were obtained from > 100 bp introns, and no correlations were detected when introns were < 100 bp.

### Divergence pattern of exons and introns

**Figure 4:**
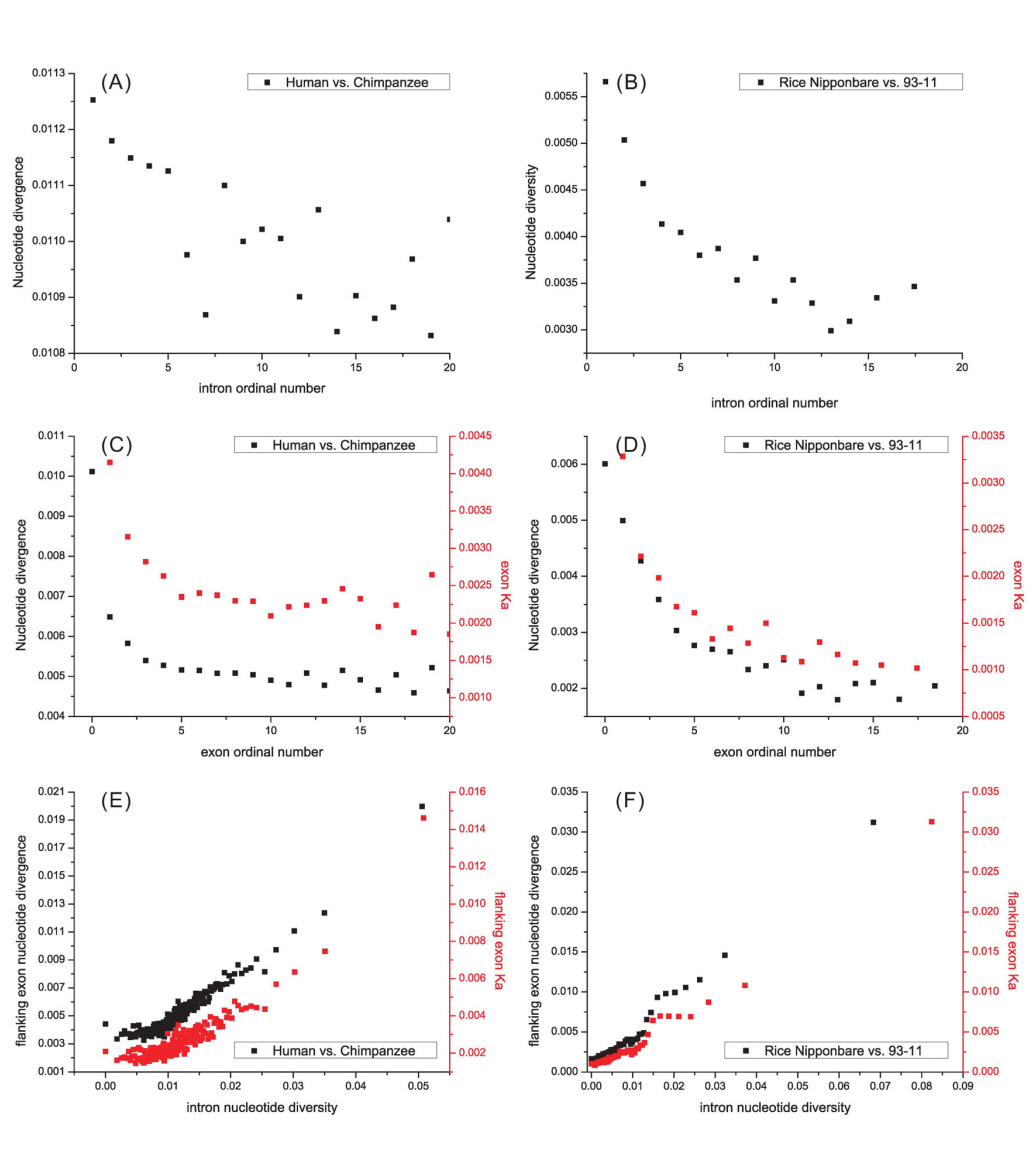
**Correlation between nucleotide divergence (or exon's Ka) and ordinal position of introns (A & B) or of exons (C & D) and between intron and flanking exon divergence (or Ka) (E & F)**. The left panel shows the results from the comparison between human and chimpanzee, and the right panel shows the comparison between two rice lines.

The divergence rate varied along intron ordinal position within genes between human and chimpanzee [7] and these same authors reported a higher level of nucleotide divergence in long introns (> 1028 bp), compared with short introns (< 1030 bp). These findings suggested that there might be more pairwise correlations among divergence, length and ordinal position in introns and exons. Therefore, we calculated all possible pairwise correlations among those traits between human and chimpanzee and between two rice cultivars. Our results confirmed that the divergence rate is strongly and negatively correlated with intron's order position not only between the human and chimpanzee but also between the two rice lines (*P *< 0.001 in both; Fig. 4A &4B & Additional file 2, Table S1). Notably, a strong negative correlation was observed between divergence and order position in exons as well in each comparison (*P *< 0.001 in both; Fig. 4C &4D). Further calculations revealed that Ka (the rate of non-synonymous substitutions) had a comparatively stronger correlation with order position (*P *< 0.001; Fig. 4C &4D) than Ks (the rate of synonymous substitutions; data not shown). A significantly positive correlation was seen between intron divergence and its flanking exon divergence or Ka (*P *< 0.001 in both; Fig. 4E &4F), indicating that when an exon has a higher level of nucleotide divergence (or Ka), a flanking intron has a high divergence as well. The strong correlations above, particularly between Ka and flanking introns, suggested that selection was involved in the high-to-low pattern of divergence as the increase of order number in both exon and intron. When an exon was under purifying selection or selective sweep, hitchhike effects could be present in its flanking introns. This effect may partly explain the close correlation of divergence between exon and flanking introns.

When the intron length was sorted by order continuously, short introns showed a lower divergence than the long introns between human and chimpanzee, and this trend was also present in the comparison of introns between the two rice lines (Additional file 1, Fig. S4A). The pattern of exon divergence was quite different from that of intron (Additional file 1, Fig. S4B). The exons, ranging from 50 to 200 bp, appear to have the lowest divergence in comparisons of both human-chimpanzee and the two rice lines.

The relationship between divergence and GC content of both exon and intron, in contrast, appears to be complicated (Additional file 1, Fig. S4C-D). In general, a positive tendency was seen between GC content and divergence in both exons and introns, in both human-chimpanzee and the two rice line comparisons. The dots shown in Additional file 1, Fig. S4C-D for each comparison significantly fit a curve line; however the mechanism behind the complicated patterns is unknown.

### Comparisons of the length, GC content and divergence betweenless-exons' and more-exons' genes

**Figure 5:**
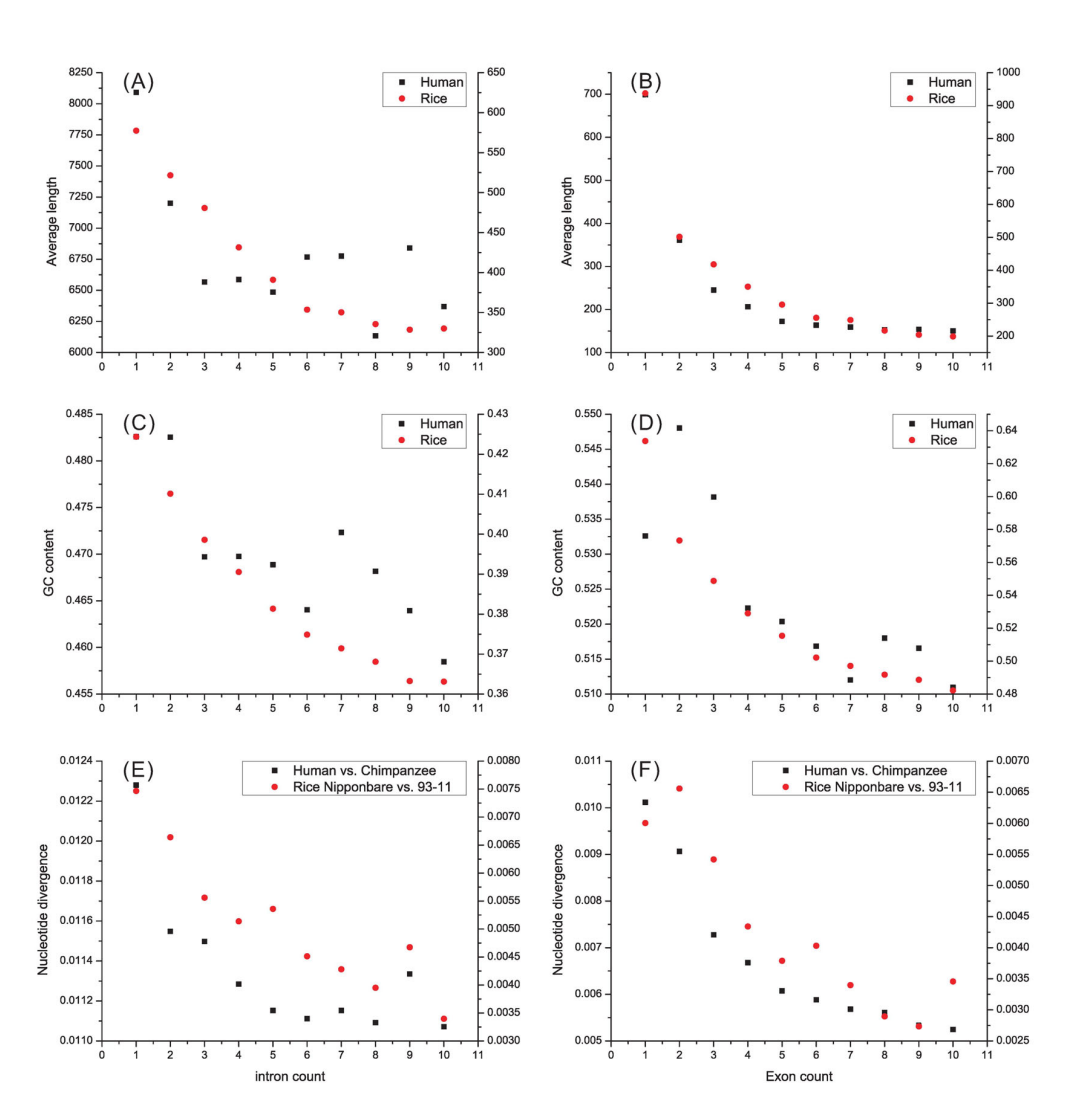
**Correlations between the length and intron (A) or exon (B) count, between GC content and intron (C) or exon (D) count and between the nucleotide divergence and intron (E) or exon (F) count**. The number (count) in the horizontal axis stands for the 1, 2-,..., 9 and 10-intron (or -exon) genes.

We further calculated the average exon (or intron) length, GC content and divergence as the increase of total exon numbers in a gene. Although the first few introns (particularly the 1^st ^– 3^rd ^introns) of the fewer-intron genes were shorter than the more-intron genes (e.g., 8091, 8397, 8888,..., 15017 bp for the first intron in the genes with 1, 2, 3,..., 10 introns in human, respectively), genes with fewer introns had relatively larger average intron length (Fig. 5A; e.g., 8091, 7200, 6567,..., 6369, bp for the genes with 1, 2, 3,..., 10 introns in human, respectively). Consistent results were also obtained for the decrease of exon length, when the last exon and UTR regions in each group of genes were excluded (Fig. 5B).

As the total number of exons or introns increases, a decrease of GC content could be seen in introns and exons of both human and rice genomes (Fig. 5C–D). In particular, the first intron (or exon) had a significantly higher GC content than the other introns (or exons). In addition, a consistent decrease of divergence was observed in both comparisons of human-chimpanzee and two rice lines as the total number of exons or introns increased (Fig. 5E–F). Notably, the decrease of exon (or intron) length, and GC content and divergence, with a corresponding increase of total number of exons (or introns), is always present in all genomes (data not shown for the other species), indicating that this is a general pattern in exon-intron variation.

## Discussion

### Basic patterns of intron-exon architecture

Our investigation of the variation patterns of exon-intron comparisons in 13 eukaryotic genomes revealed three basic patterns: an ordinal reduction of length and divergence in both exon and intron (Fig. 2 and 4A–D); a co-variation of GC content and divergence between exons and flanking introns (Fig. 3 and 4E–F); and a decrease of average exon (or intron) length, GC content and divergence as the increase of total exon numbers in a gene increased. The three basic patterns existed in almost all genomes analyzed and showed strong correlation (*P *< 0.001 normally) with a generally consistent variation pattern. In addition, a strong complicated correlation (*P *< 0.001 normally) exists between GC content and the length of introns (or exons), and this correlation is present in all genomes analyzed. Although more significant correlations were observed (e.g., between divergence and length of exons or introns), only these four patterns were consistent among species.

To assess the reliability of our observations, we did many different calculations for each pattern. For example, we calculated the length, GC content or divergence separately for the genes having different numbers of introns (or exons) for the pattern of ordinal reduction in introns and exons. In other words, we calculated the two-intron, three-intron... 9-intron and > 9-intron (or -exon) genes separately, to compare the genes with an equal number of introns or exons. We chose human and rice (or human *vs. *chimpanzee and rice Nipponbare *vs. *93-11) as examples to assess the consistency (Additional file 1, Fig. S5 – S6). Indeed, our calculations confirmed that the basic patterns observed are reliable, although those depicted in Figs. 4B and 4D are not always present in the gene groups with equal number of exons or introns in the comparison of two rice lines.

### Mechanisms and evolutionary forces underlying the basic variation patterns

The first systematic analysis of 13 eukaryotic species revealed there are three general patterns of exon-intron structure variation. Although the mechanisms (or factors) that cause these patterns remain unclear, our results provide some clues to such a phenomenon. Firstly, the co-variation pattern of exon-intron in their GC content, length and divergence suggests that the basic variation patterns are caused by factor(s) common to either exons or introns or to both. Given that there are common factors, they are likely to affect exons and functional elements of introns because of their importance. Therefore, the functional association between exon and intron, e.g., the splicing elements, may be the basis of co-variation patterns. Secondly, the monotonic reduction of length, GC content and divergence as the ordinal variation or as the total number increase in introns or exons indicates that this pattern may be the key to decipher the major factors which shape these patterns. The decay-like curves for these orderly reductions suggest that the ordinal patterns may be a reflection of time-related order (e.g., the longer or the more divergent introns occurred earlier). Thirdly, the first and the last exons are generally the longest exons in a gene, indicating the necessity of maintaining a larger exon in the beginning and in the end of a coding sequence. The selectively maintained first and last larger exons may be transcription-, splicing- or translation-required. Finally, the first intron generally has the highest GC content and thereafter there is almost no regular ordinal-pattern, which suggests that the first intron may evolve differently from the other introns.

In fact, the enrichment of regulatory elements in the first intron was most often used to explain the evolutionary pattern of the intron [7]. For example, the first intron was shown to enhance gene expression more than any others [23,24]. If the first intron is enriched with regulatory elements, they should thus contain more GC nucleotides, consistent with the higher GC content observed in most of genomes analyzed. However, the possible enrichment of regulatory elements in the first intron alone cannot explain the basic patterns in exon-intron variation as well as co-variation patterns between exons and introns.

The exon-intron architecture was also revealed to be important in the determination of splicing phenotype, and the efficient recognition of exons was influenced by the length of flanking introns [25]. The splice sites are recognized across the exons when intron size is greater than 200 to 250 nucleotides [26]. The intron size has a profound influence on the likelihood that an exon is constitutively or alternatively spliced. Exon skipping is more likely to occur when exons are flanked by long introns in the human genome [25,26]. Intron and exon lengths within a genome can reflect the constraints imposed by splicing [27]. Many other factors may also contribute to the intron evolution, such as the density of transposable elements [13], the attendance of RNA genes [15] or miRNA [8] and the frequency and size of insertion/deletion events [14]. Similar to the hypothesis of enrichment of regulatory elements in the first introns, however, these factors also cannot be used to explain either the ordinal or co-variation pattern in both exons and introns. Marais *et al*. [28] suggested that genes with more slowly evolving exon sequences might also have more regulatory elements in introns. In this hypothesis, a co-variation pattern of exon-introns is expected but the strong ordinal reduction is not anticipated.

Obviously, the ordinal (or orderly) reduction patterns of exon-intron architecture constitute an important puzzle with theories about the determinants of genome evolution. The ordinal patterns may reflect a time-orderly evolution. If the amount of exons or introns follows an increasing trend (during the process of genome-size expansion), the first exon and intron are older than the next ones; the older introns have more time to be inserted and become longer; the inserted sequences in introns generally have a lower GC content; and the later occurring introns cut the coding sequences into shorter ones except for the first and the last exon, which are required by splicing-related factors; the subsequent recruited exons (or parts of them), compared with the first exons, have a higher possibility of coming from intron sequences and therefore have a lower GC content. The timely-ordered model could describe the consistent decrease of length, GC content and divergence observed in this study.

Some expectations could be tested by comparing the side (the first or the last) and the inner exons. Based on the UniProt database [29], the proportion of the alternative spliced human exons in the total number of exons could be calculated separately for the genes with 3, 4... 9, and > 9-exons, respectively. Then we could compare the proportions of the first exon, the last exon and the others between them. Our calculation showed that the first (3.31%) and the last exon (5.98%) had a higher proportion than 2.33% in the others, indicating that the first and the last exon tend to be spliced alternatively, which might contribute to their unique lengths or GC contents.

The exon-increase model involves more in the variation of existed exons/introns, e.g., their length, divergence and GC content, but less in their gain and loss. Under this model, the preferential gain of introns in 3'-portions of genes is expected. This is more consistent with the introns-late hypothesis, in which the introns appeared later and at random in early eukaryotic genomes, and gained an adaptive role in gene evolution following insertion [30]. Sverdlov and coworkers [31] compared the distributions of old and new introns along the length of eukaryotic genes and showed that old introns were substantially overrepresented in the 5'-portions of the genes in all sequenced eukaryotic genomes, while new introns were over-distributed in the 3'-regions of the genes in the most intron-rich genomes. These results could be interpreted as an indication that the introns in the 3'-regions of genes might be younger. Similar to intron late or early hypothesis, however, this model cannot explain why the number of exons or introns increased in some genes or genomes but not in the others, neither does it define the outcome of this process. Many fundamental questions remain to be addressed in further studies.

## Conclusion

We showed that there were three basic patterns of exon-intron variation in eukaryotic genomes, and these patterns were consistently present in almost all 13 genomes analyzed. The first pattern is the ordinal reduction of length and divergence in both exons and introns. The decay-curve-like variation and the orderly reduction suggest that this pattern may reflect a time-sequential evolution (e.g., the earlier-occurred introns or exons were longer or more divergent). The second pattern is the co-variation of length, GC content and divergence between exons and flanking introns. The closely-correlated co-variation between exons and introns indicates common factor(s) exist in shaping both exons and introns. The third pattern is the decrease of average length, GC content and divergence in exons and introns as the total number of exons in a gene increased. This phenomenon may also be a reflection of chronological variation. In addition, a significant non-linear correlation was observed between GC content and the length of introns or exons. All these patterns of exon-intron variation revealed by our investigation provide a framework for elucidating mechanisms involved in the organization of eukaryotic genomes and particularly in building exon-intron structures.

## Methods

### Genome sequences

Thirteen full sequenced genomes were selected (Table 1), including Human (*Homo sapiens*), chimpanzee (*Pan troglodytes*), mouse (*Mus musculus*), rat (*Rattus norvegicus*), Cow (*Bos taurus*), dog (*Canis familiaris*), chicken (*Gallus gallus*), zebrafish (*Danio rerio*), pufferfish (*Tetraodon nigroviridis*), fruit fly (*Drosophila melanogaster*), worm (*Caenorhabditis elegans*), *Arabidopsis thaliana *and rice (*Oryza Sativa*). The genome sequences and annotations of two rice lines (*Oryza sativa *L. var. Nipponbare vs. var. 93-11) were obtained from GRAMENE [32]. All genes and gene structures for other genomes were identified and retrieved from Ensembl (build 44) [33]. Ensembl's Perl modules (build 44) [34] were used to obtain exon and intron lengths for all genes. A local MySQL database was built to store all relevant information of each intron and exon, including length, GC content, intron order number and coverage of repeat elements. The sequences for each trait were retrieved using Ensembl's Perl modules and were stored locally.

### Analysis of exons and introns

To investigate the relationships among length, GC content, ordinal position and nucleotide divergence in both introns and exons, each trait was first sorted by order, and then partitioned into bins containing 1000 introns or 1000 exons each (shown as the x axis in all figures). Then another trait (y axis) was calculated for each of these bins and plotted against the first trait. For example, when examining the relationship between intron length and GC content, all introns in a genome were first sorted either by length or GC content into bins, then the average of GC content or length of every bin was calculated for each bin and plotted against length or GC content in figures. Only those introns within coding sequences and exons without UTR (untranslated regions) were used for analyses in all figures. In addition, the short introns (< 20 bp) were excluded to avoid systematic errors, which were reported in the introns by using automated annotation methods [10].

### Sequence alignment and divergence calculation

The pairwise alignments between human and chimpanzee were downloaded directly from UCSC Genome Browser [35]. Pairwise alignments between the two rice lines were performed by BlastZ [36]. The C-language source code for BlastZ and the code for extracting lineage-specific repeats are publicly available [37]. The BlastZ scoring matrix was the same as the one UCSC used for the pairwise alignments of human and chimpanzee sequence. For every intron or exon, the nucleotide divergence was measured applying the Jukes-Cantor correction to the number of substitutions per site.

## Authors' contributions

DT and JQC designed the study and drafted the manuscript. LZ and YZ contributed extensively to the bioinformatics analyses. WZ and SY assisted the analyses and manuscript preparation. All authors read and approved the final manuscript.

## Supplementary Material

Additional file 1 **Fig S1-6.** The other relationship between the length, GC content, divergence of introns/exons. Click here for file

Additional file 2 **Table S1-2.** The statistical test results. Click here for file
