# Supplementary material for: Patterns of exon-intron architecture variation of genes in eukaryotic genomes
Source: BMC Genomics. 2009 Jan 24;10:47. doi: 10.1186/1471-2164-10-47 (PMC2636830; doi:10.1186/1471-2164-10-47)
Supplement: Additional file 1 — Fig S1-6. The other relationship between the length, GC content, divergence of introns/exons. [file 1471-2164-10-47-S1.doc]

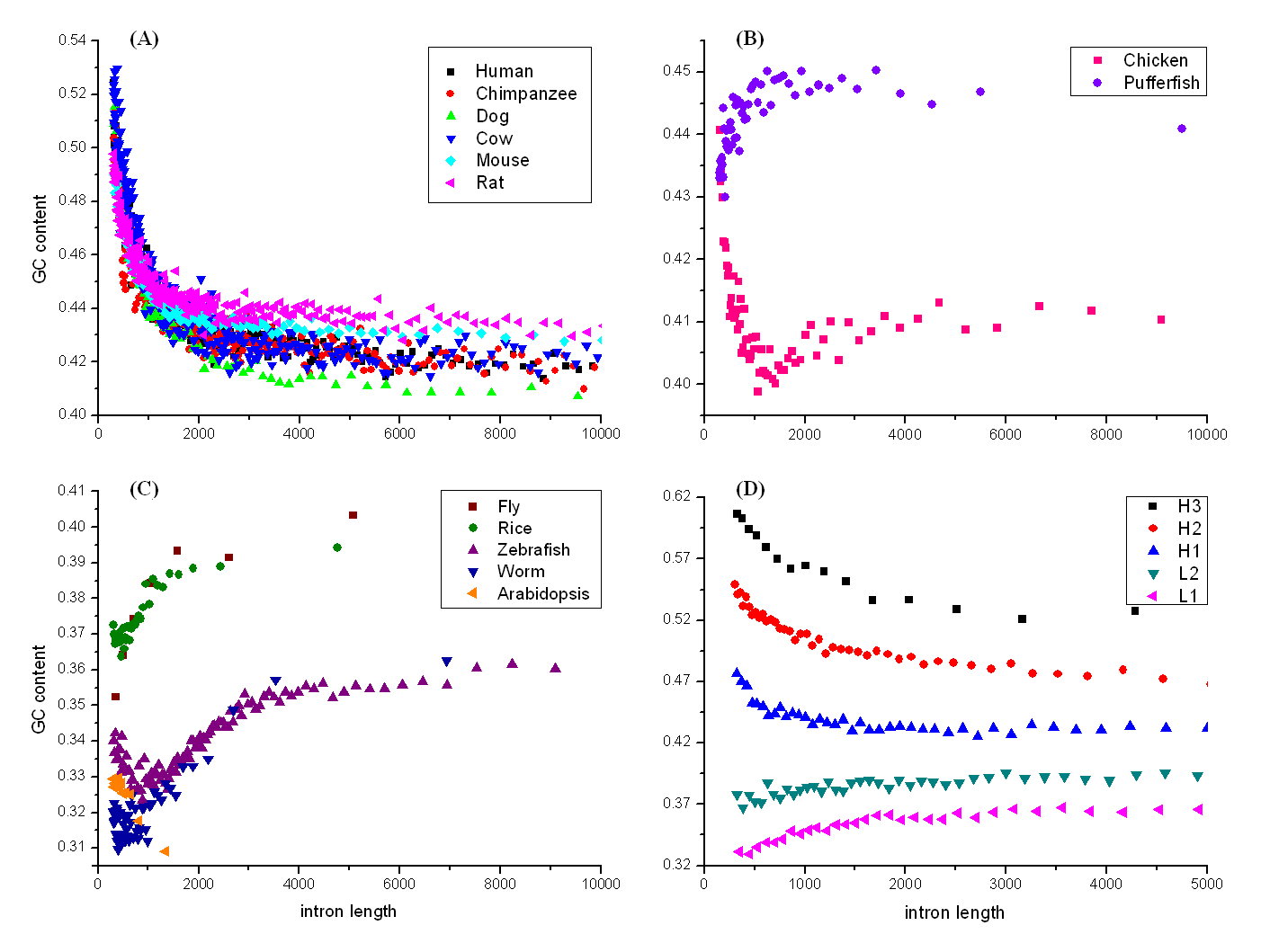


Supplementary Figure 1. Variation patterns of introns’ GC content, as a function of intron length (300~10000 bp) (A-D). L1, L2, H1, H2 and H3 in D represent five GC-isochore families in human genome (Costantini et al 2006) which are different in GC-content (0~0.37, 0.37~0.41, 0.41~0.46, 0.46~0.53, 0.53~1, respectively). Genes that cover two or more family regions were excluded in D. The introns in each class were sorted by length and then each 1000 introns were combined as one dot.


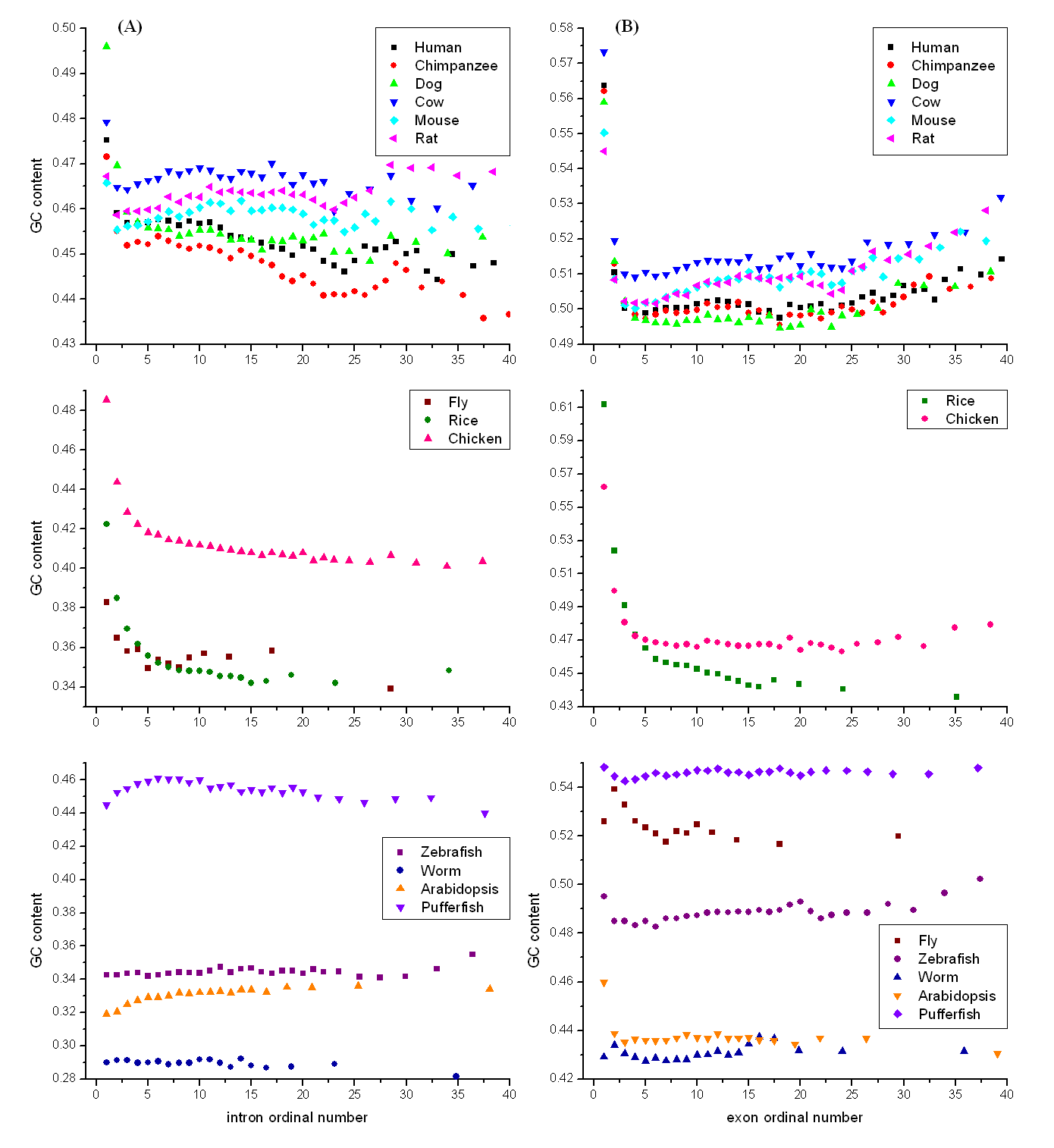


Supplementary Figure 2. Correlation between the GC content and intron (A) or exon ordinal position (B).


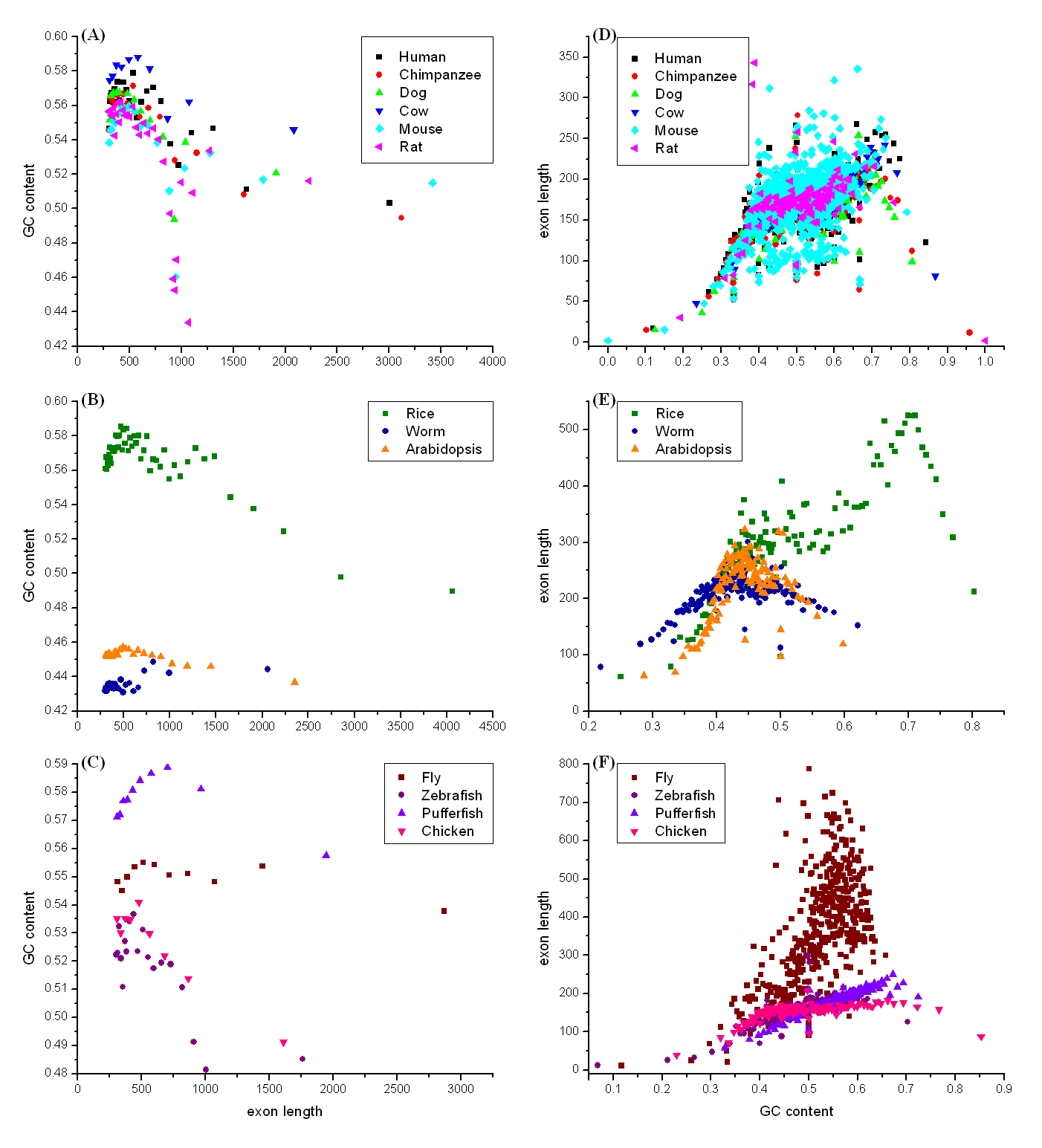


Supplementary Figure 3. Variation patterns of exons’ GC content, as a function of exon length (>300 bp) (left); or patterns of exons’ length (>300 bp)*,* as a function of GC content (right). Each dot contains 1000 exons.


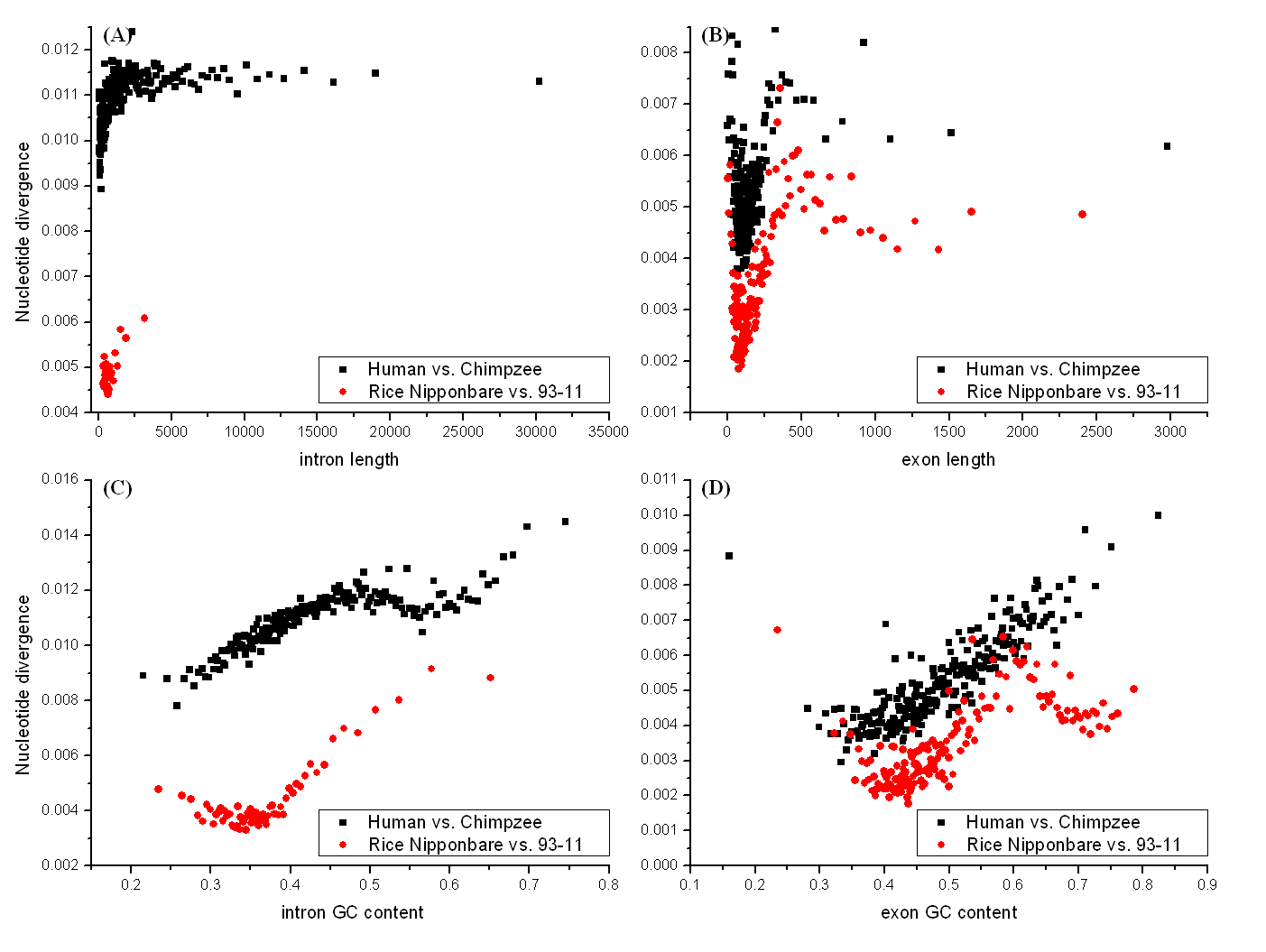


Supplementary Figure 4. Correlations between divergence and length in introns (A) or exons (B) and between divergence and GC content in introns (C) or exons (D).


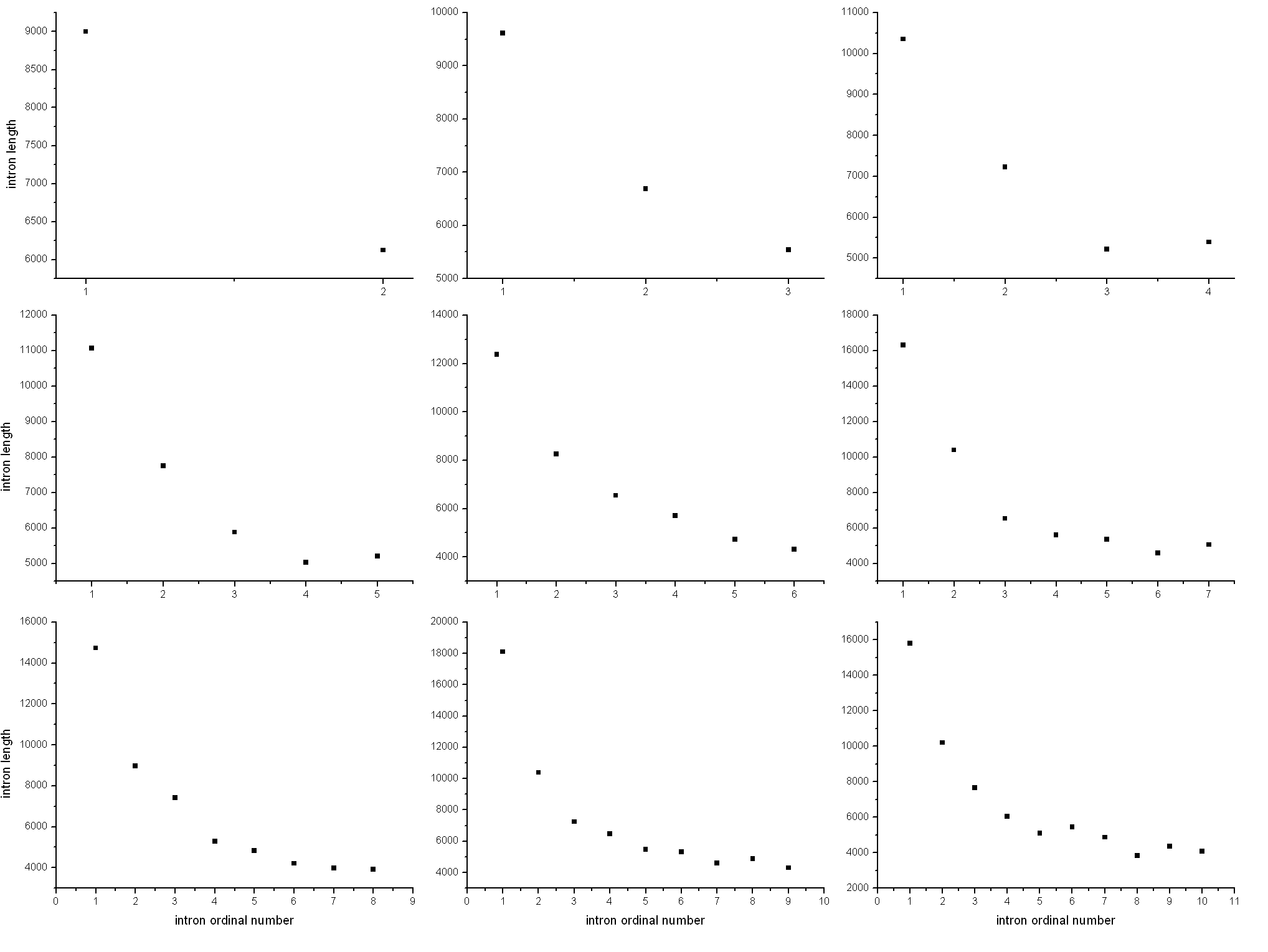


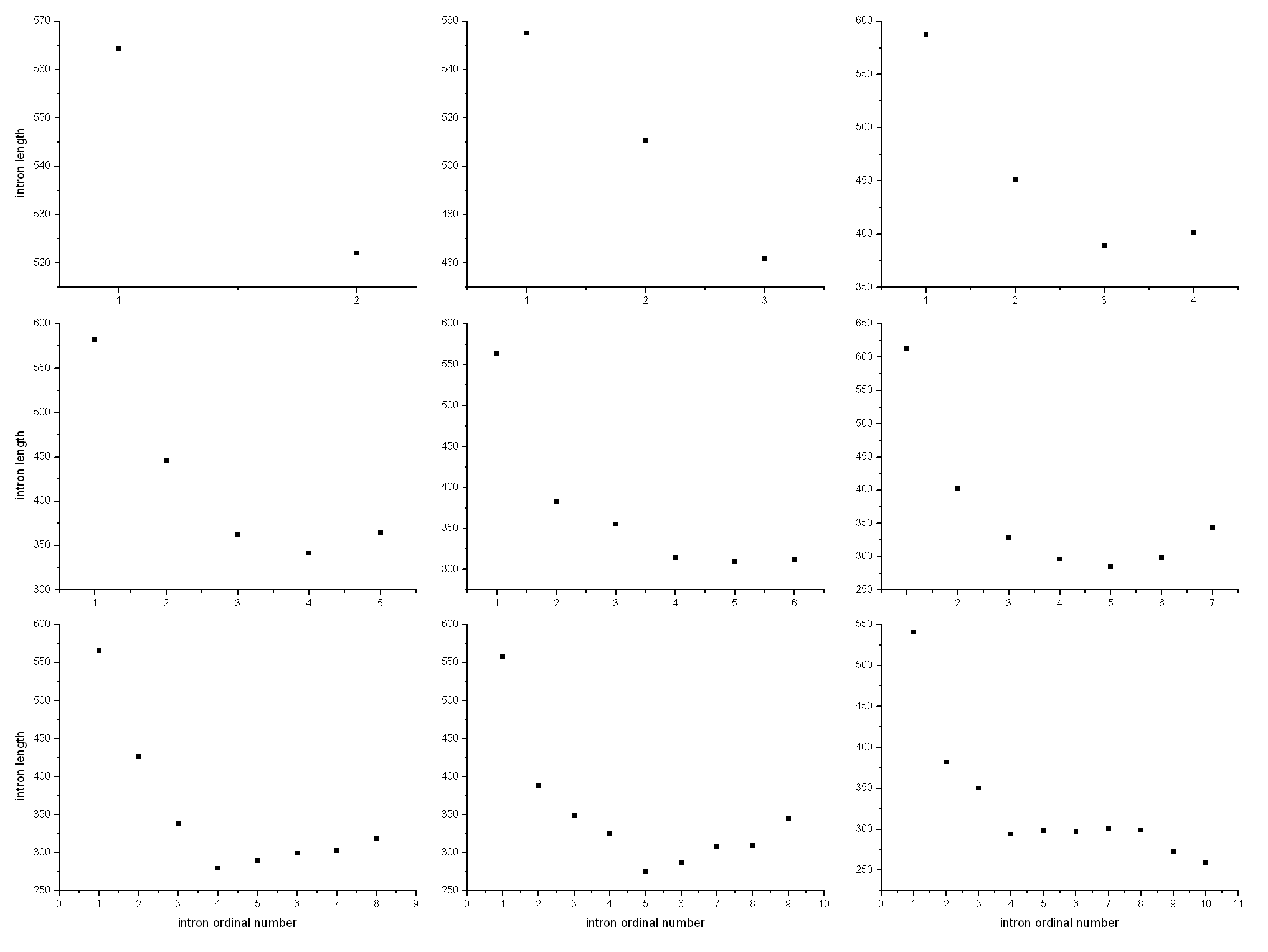


Supplementary Figure 5. The ordinal reduction of length in human genome (top 9 figures) and in rice genome (bottom 9 figures) when comparing the genes with the same number of introns. Each dot contains >1000 in human and >500 exons in rice.


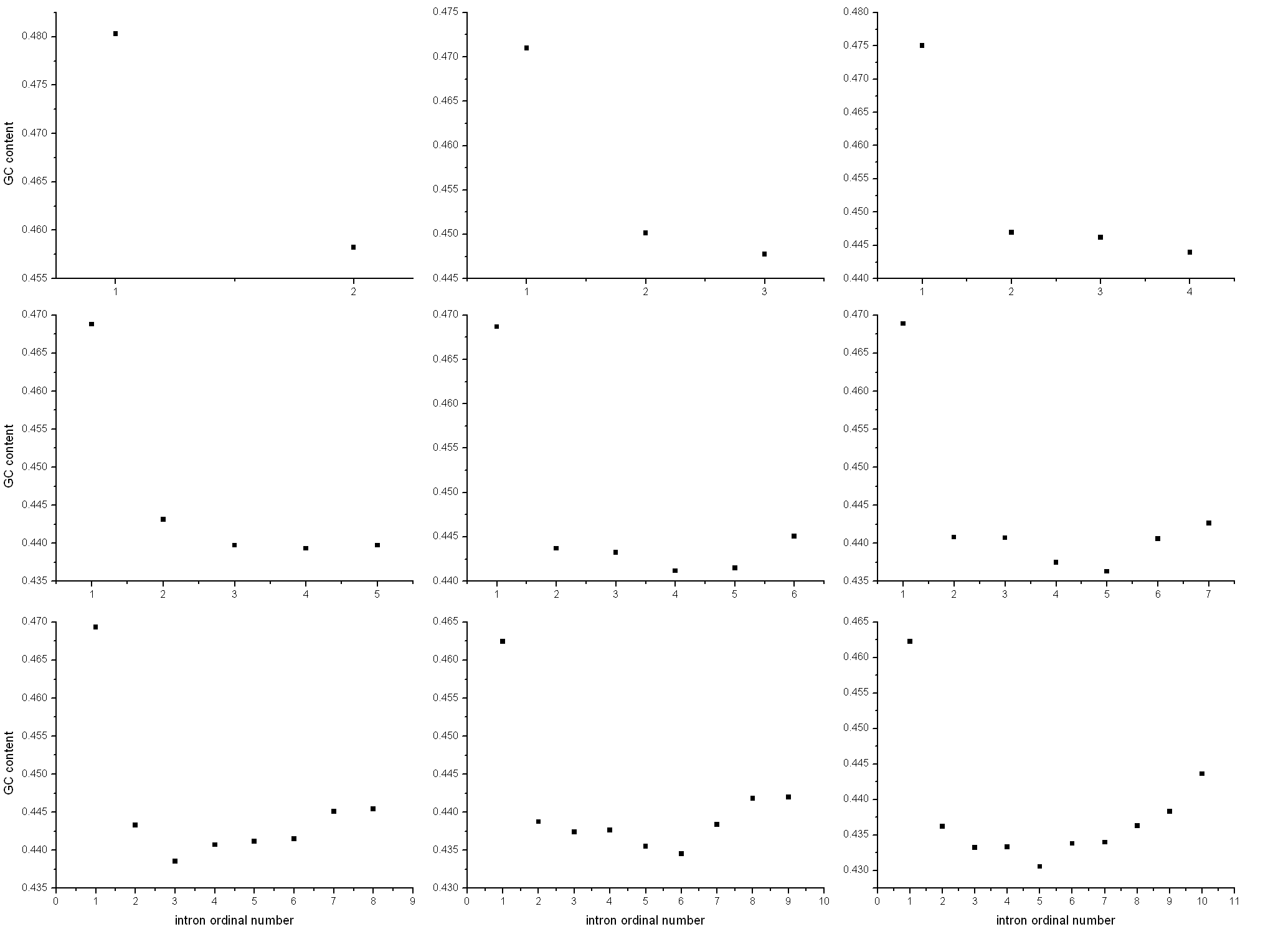


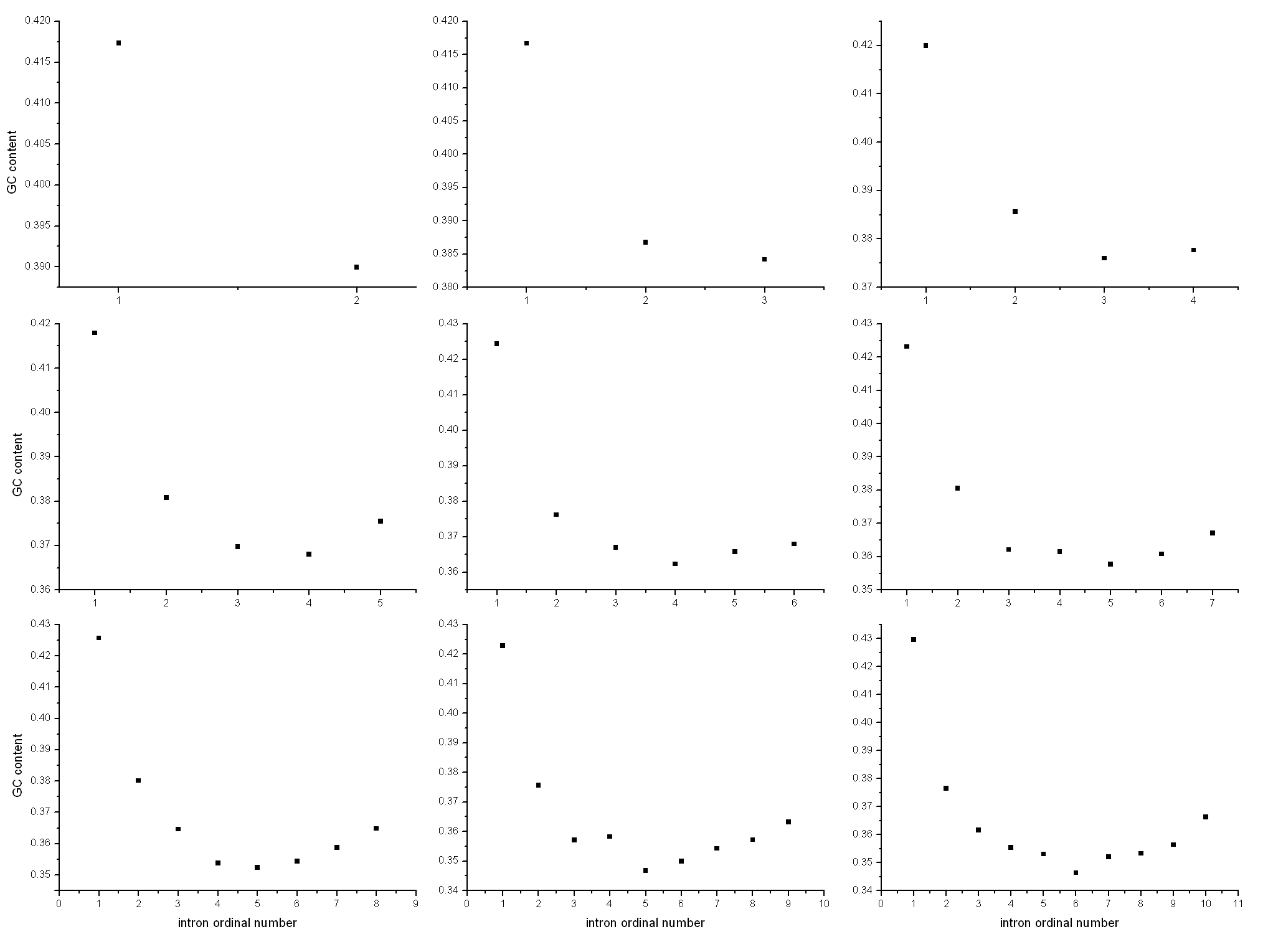


Supplementary Figure 6. The ordinal reduction of GC-content in human genome (top 9 figures) and in rice genome (bottom 9 figures) when comparing the genes with the same number of introns. Each dot contains >1000 in human and >500 exons in rice.
