# Supplementary material for: Patterns of exon-intron architecture variation of genes in eukaryotic genomes
Source: BMC Genomics. 2009 Jan 24;10:47. doi: 10.1186/1471-2164-10-47 (PMC2636830; doi:10.1186/1471-2164-10-47)
Supplement: Additional file 2 — Table S1-2. The statistical test results. [file 1471-2164-10-47-S2.doc]

Supplementary table 1: A list of *P*-values for significance test

| Fig | Name | intron count^1^ | R | P-value |
| --- | --- | --- | --- | --- |
|
| Fig. S1A | Human |  | 0.94 | 0 |
| Fig. S1A | Chimpanzee |  | 0.94 | 0 |
| Fig. S1A | Dog |  | 0.95 | 0 |
| Fig. S1A | Cow |  | 0.91 | 0 |
| Fig. S1A | Mouse |  | 0.94 | 0 |
| Fig. S1A | Rat |  | 0.94 | 0 |
| Fig. S1B | Pufferfish |  | 0.7 | 0 |
| Fig. S1B | Chicken |  | 0.69 | 0 |
| Fig. S1C | Fly |  | 0.98 | 2.12E-10 |
| Fig. S1C | Rice |  | 0.92 | 0 |
| Fig. S1C | Zebrafish |  | 0.85 | 0 |
| Fig. S1C | Worm |  | 0.88 | 0 |
| Fig. S1C | Arabidopsis |  | 0.97 | 1.66E-08 |
| Fig.1A | Human |  | 0.66 | 0 |
| Fig.1A | Chimpanzee |  | 0.67 | 0 |
| Fig.1A | Dog |  | 0.64 | 0 |
| Fig.1A | Cow |  | 0.65 | 0 |
| Fig.1A | Mouse |  | 0.75 | 0 |
| Fig.1A | Rat |  | 0.71 | 0 |
| Fig.1B | Zebrafish |  | 0.62 | 0 |
| Fig.1B | Fly |  | 0.61 | 7.98E-11 |
| Fig.1B | Chicken |  | 0.64 | 0 |
| Fig.1C | Rice |  | 0.81 | 0 |
| Fig.1C | Worm |  | 0.79 | 0 |
| Fig.1C | Arabidopsis |  | 0.46 | 0 |
| Fig.1C | Pufferfish |  | 0.7 | 0 |
| Fig.2A | Human |  | 0.97 | 0 |
| Fig.2A | Chimpanzee |  | 0.97 | 0 |
| Fig.2A | Dog |  | 0.97 | 0 |
| Fig.2A | Cow |  | 0.98 | 0 |
| Fig.2A | Mouse |  | 0.98 | 0 |
| Fig.2A | Rat |  | 0.97 | 0 |
| Fig.2A | Arabidopsis |  | 0.95 | 0 |
| Fig.2A | Fly |  | 0.97 | 2.10E-09 |
| Fig.2A | Zebrafish |  | 0.98 | 0 |
| Fig.2A | Chicken |  | 0.98 | 0 |
| Fig.2A | Rice |  | 0.96 | 0 |
| Fig.2A | Worm |  | 0.68 | 0 |
| Fig.2A | Pufferfish |  | 0.96 | 0 |
| Fig.S2A | Fly |  | 0.89 | 0 |
| Fig.S2A | Rice |  | 0.97 | 0 |
| Fig.S2A | Chicken |  | 0.99 | 0 |
| Fig.2B | Human |  | 0.59 | 0 |
| Fig.2B | Chimpanzee |  | 0.52 | 0 |
| Fig.2B | Dog |  | 0.88 | 0 |
| Fig.2B | Cow |  | 0.82 | 0 |
| Fig.2B | Mouse |  | 0.73 | 0 |
| Fig.2B | Rat |  | 0.89 | 0 |
| Fig.2B | Arabidopsis |  | 0.93 | 0 |
| Fig.2B | Fly |  | 0.65 | 0.01715 |
| Fig.2B | Zebrafish |  | 0.71 | 0 |
| Fig.2B | Chicken |  | 0.79 | 0 |
| Fig.2B | Rice |  | 0.97 | 0 |
| Fig.2B | Worm |  | 0.88 | 4.99E-07 |
| Fig.2B | Pufferfish |  | 0.35 | 0 |
| Fig. S3D | Human |  | 0.6 | 0 |
| Fig. S3D | Chimpanzee |  | 0.57 | 0 |
| Fig. S3D | Dog |  | 0.71 | 0 |
| Fig. S3D | Cow |  | 0.77 | 0 |
| Fig. S3D | Mouse |  | 0.44 | 0 |
| Fig. S3D | Rat |  | 0.55 | 0 |
| Fig. S3E | Fly |  | 0.59 | 0 |
| Fig. S3E | Zebrafish |  | 0.76 | 0 |
| Fig. S3E | Pufferfish |  | 0.92 | 0 |
| Fig. S3E | Chicken |  | 0.82 | 0 |
| Fig. S3F | Rice |  | 0.83 | 0 |
| Fig. S3F | Worm |  | 0.76 | 0 |
| Fig. S3F | Arabidopsis |  | 0.75 | 0 |
| Fig.3A | Human |  | 0.98 | 0 |
| Fig.3A | Chimpanzee |  | 0.97 | 0 |
| Fig.3A | Dog |  | 0.98 | 0 |
| Fig.3A | Cow |  | 0.97 | 0 |
| Fig.3A | Mouse |  | 0.97 | 0 |
| Fig.3A | Rat |  | 0.97 | 0 |
| Fig.3A | Chicken |  | 0.99 | 0 |
| Fig.3B | Fly |  | 0.96 | 0 |
| Fig.3B | Zebrafish |  | 0.97 | 0 |
| Fig.3B | Rice |  | 0.97 | 0 |
| Fig.3B | Pufferfish |  | 0.99 | 0 |
| Fig.3C | Worm |  | 0.89 | 0 |
| Fig.3C | Arabidopsis |  | 0.76 | 0 |
| Fig.3D | Human |  | 0.67 | 0 |
| Fig.3D | Chimpanzee |  | 0.7 | 0 |
| Fig.3D | Dog |  | 0.82 | 0 |
| Fig.3D | Cow |  | 0.87 | 0 |
| Fig.3D | Mouse |  | 0.72 | 0 |
| Fig.3D | Rat |  | 0.83 | 0 |
| Fig.3D | Chicken |  | 0.89 | 0 |
| Fig.3E | Rice |  | 0.6 | 2.39E-08 |
| Fig.3E | Arabidopsis |  | 0.95 | 0 |
| Fig.3E | Zebrafish |  | 0.86 | 0 |
| Fig.3E | Pufferfish |  | 0.46 | 6.61E-10 |
| Fig.3F | Fly |  | 0.61 | 2.54E-06 |
| Fig.3F | Worm |  | 0.27 | 0.00327 |
| Fig.4A |  |  | 0.73 | 2.85E-04 |
| Fig.4B |  |  | 0.85 | 3.72E-05 |
| Fig.4C |  |  | 0.92 | 0 |
| Fig.4D |  |  | 0.98 | 0 |
| Fig.4E |  |  | 0.96 | 0 |
| Fig.4F |  |  | 0.99 | 0 |
| Fig.5A | Human |  | 0.9 | 1.86E-10 |
| Fig.5A | Rice |  | 0.99 | 8.70E-11 |
| Fig.5B | Human |  | 0.99 | 2.48E-08 |
| Fig.5B | Rice |  | 1 | 6.47E-12 |
| Fig.5C | Human |  | 0.87 | 5.88E-15 |
| Fig.5C | Rice |  | 1 | 0 |
| Fig.5D | Human |  | 0.85 | 1.25E-13 |
| Fig.5D | Rice |  | 1 | 1.11E-16 |
| Fig.5E | Human |  | 0.96 | 9.10E-15 |
| Fig.5E | Rice |  | 0.96 | 1.31E-08 |
| Fig.5F | Human |  | 0.98 | 6.88E-10 |
| Fig.5F | Rice |  | 0.93 | 4.43E-07 |
| Fig.S5 | Human | 4 | 0.99 | 0.05908 |
| Fig.S5 | Human | 5 | 0.99 | 0.00258 |
| Fig.S5 | Human | 6 | 1 | 2.32E-06 |
| Fig.S5 | Human | 7 | 0.99 | 7.50E-05 |
| Fig.S5 | Human | 8 | 1 | 4.19E-07 |
| Fig.S5 | Human | 9 | 1 | 4.01E-08 |
| Fig.S5 | Human | 10 | 0.99 | 1.12E-08 |
| Fig.S5 | Rice | 4 | 0.99 | 0.03317 |
| Fig.S5 | Rice | 5 | 0.98 | 0.00279 |
| Fig.S5 | Rice | 6 | 1 | 1.45E-05 |
| Fig.S5 | Rice | 7 | 0.97 | 5.43E-05 |
| Fig.S5 | Rice | 8 | 0.95 | 6.72E-06 |
| Fig.S5 | Rice | 9 | 0.95 | 3.78E-07 |
| Fig.S5 | Rice | 10 | 0.99 | 3.95E-10 |
| Fig.S6 | Human | 4 | 1 | 0.00124 |
| Fig.S6 | Human | 5 | 1 | 3.20E-06 |
| Fig.S6 | Human | 6 | 0.99 | 4.90E-08 |
| Fig.S6 | Human | 7 | 0.98 | 1.01E-09 |
| Fig.S6 | Human | 8 | 0.97 | 1.19E-11 |
| Fig.S6 | Human | 9 | 0.95 | 1.09E-13 |
| Fig.S6 | Human | 10 | 0.91 | 1.04E-14 |
| Fig.S6 | Rice | 4 | 0.99 | 0.00583 |
| Fig.S6 | Rice | 5 | 0.98 | 1.43E-04 |
| Fig.S6 | Rice | 6 | 0.99 | 8.39E-07 |
| Fig.S6 | Rice | 7 | 0.97 | 7.60E-08 |
| Fig.S6 | Rice | 8 | 0.96 | 3.79E-09 |
| Fig.S6 | Rice | 9 | 0.96 | 1.01E-10 |
| Fig.S6 | Rice | 10 | 0.96 | 2.74E-12 |
| intron D_value | Human | 7 | 0.85 | 7.82E-09 |
| intron D_value | Human | 8 | 0.8 | 1.31E-09 |
| intron D_value | Human | 10 | 0.46 | 5.59E-12 |
| exon_D_value | Human | 4 | 1 | 0.03846 |
| exon_D_value | Human | 5 | 0.97 | 0.0344 |
| exon_D_value | Human | 6 | 0.98 | 3.01E-04 |
| exon_D_value | Human | 7 | 0.99 | 1.48E-06 |
| exon_D_value | Human | 8 | 0.83 | 4.23E-06 |

1. This column is useful when the gene were classified by the intron count they contains.

Supplementary table 2:

| Fig | Name | GC^1^(%) | GC^2^(%) | P_vlaue |
| --- | --- | --- | --- | --- |
|
| Fig.S2A | Human | 47.53 | 45.47 | 7.10E-265 |
| Fig.S2A | Chimpanzee | 47.16 | 44.95 | 2.05E-202 |
| Fig.S2A | Dog | 49.60 | 45.61 | 3.71E-211 |
| Fig.S2A | Cow | 47.93 | 46.65 | 4.89E-42 |
| Fig.S2A | Mouse | 46.58 | 45.78 | 1.98E-48 |
| Fig.S2A | Rat | 46.73 | 46.18 | 2.09E-18 |

1. The average GC-content of the first introns
2. The average GC-content of the introns except the first ones.
